# Multiple Sensors Data Integration for Traffic Incident Detection Using the Quadrant Scan

**DOI:** 10.3390/s22082933

**Published:** 2022-04-11

**Authors:** Ayham Zaitouny, Athanasios D. Fragkou, Thomas Stemler, David M. Walker, Yuchao Sun, Theodoros Karakasidis, Eftihia Nathanail, Michael Small

**Affiliations:** 1 ARC Training Centre for Transforming Maintenance Through Data Science, University of Western Australia, 35 Stirling Highway, Crawley, WA 6009, Australia; michael.small@uwa.edu.au; 2 Complex Systems Group, Department of Mathematics and Statistics, University of Western Australia, 35 Stirling Highway, Crawley, WA 6009, Australia; thomas.stemler@uwa.edu.au (T.S.); david.walker@uwa.edu.au (D.M.W.); 3 Department of Civil Engineering, University of Thessaly, 38334 Volos, Greece; fthanos@uth.gr (A.D.F.); thkarak@uth.gr (T.K.); enath@uth.gr (E.N.); 4 Planning and Transport Research Centre (PATREC), University of Western Australia, 35 Stirling Highway, Crawley, WA 6009, Australia; chao.sun@uwa.edu.au; 5 Department of Physics, University of Thessaly, 35100 Lamia, Greece

**Keywords:** traffic monitoring, traffic management, non–recurrent congestion, major/minor incident, incident detection, recurrence plots, Quadrant Scan

## Abstract

Non-recurrent congestion disrupts normal traffic operations and lowers travel time (TT) reliability, which leads to many negative consequences such as difficulties in trip planning, missed appointments, loss in productivity, and driver frustration. Traffic incidents are one of the six causes of non-recurrent congestion. Early and accurate detection helps reduce incident duration, but it remains a challenge due to the limitation of current sensor technologies. In this paper, we employ a recurrence-based technique, the Quadrant Scan, to analyse time series traffic volume data for incident detection. The data is recorded by multiple sensors along a section of urban highway. The results show that the proposed method can detect incidents better by integrating data from the multiple sensors in each direction, compared to using them individually. It can also distinguish non-recurrent traffic congestion caused by incidents from recurrent congestion. The results show that the Quadrant Scan is a promising algorithm for real-time traffic incident detection with a short delay. It could also be extended to other non-recurrent congestion types.

## 1. Introduction

Congestion causes travel delays that result in significant economic costs to road users [1]. For example, in 2018, an average vehicle in the UK had spent over 40 h in congestion, more than the number of working hours in a week [2]. There is no universal definition of congestion [3], but we adopt the one that is more aligned with traffic engineering principles—congestion happens when demand exceeds capacity [4,5]. There are two types of congestion, recurrent and non-recurrent. The former pertains to regular occurrences of having volumes higher than the base capacity, which commonly happen during peak hours. Non-recurrent congestion caused by incidents is characterized by temporary volume-over-capacity occurrences with irregular triggers that either cause an increase in volume or decrease in capacity. Potts et al. (2014) categorize them into six groups, i.e., traffic incidents, severe weather, special events, work zones, demand fluctuations, and traffic control devices [6]. Non-recurrent congestion disrupts normal traffic operations and lowers travel time (TT) reliability which leads to many negative consequences, such as difficulties in trip planning, missed appointments, loss in productivity, and driver frustration (ibid).

This paper focuses on one of the most common causes of non-recurrent congestion—traffic incidents, more specifically, the detection of them. Traffic incidents include vehicle mechanical failure, flat tyres, debris on the roadway, crashes, etc. [7,8]. In the U.S., they are estimated to be accountable for about 25% of total congestion [9] and the number was estimated to be about 15% for the Central London Area [10].

Incident duration is composed of three phases: detection/verification, response, and clearance [11]. Some authors also include recovery time as the fourth phase [12,13]. Reducing incident duration is crucial in minimizing its negative impacts, with every minute of reduction estimated to save each motorist five minutes for off-peak incidents. The number would be higher during peak traffic [14]. Data collected in Washington State in 1994 and 1995 showed that the first phase took an average of about 12 min [11]. Meanwhile, a study using data from the Utah Department of Transportation concluded that every minute of delay in response time added an average of $925 to excess user costs and 93 more affected vehicles in 2018 [15]. The actual numbers must differ between jurisdictions, but early and accurate detection will certainly reduce the time spent on verification and contribute to the reduction of incident duration.

Despite the large amount of existing literature, incident detection is still an active field of research, which in our opinion is largely due to the limitations of sensor technologies. There are many types of sensors on the market, such as inductive loops, magnetometers, pneumatic tubes, piezoelectric sensors, radar, Lidar, infrared devices, and traffic cameras, which can be divided into three categories: roadway-based, probe-based and driver-based [16]. Each technology has its own weaknesses in accuracy, sample size, types of data collected, and coverage etc. [17]. More importantly, most sensors are designed to collect traffic data, such as speed, flow, and density, which are incidental measures of traffic incidents. Therefore, algorithms are required to make the inference, which always comes with a degree of uncertainty that needs to be verified by humans. Video cameras are the best option because they can be used for manual observation. However, very few jurisdictions (if any) would have a complete coverage of the entire network, so most agencies have to rely on other types of sensors and apply incident-detection algorithms.

The algorithms developed so far fall into three broad categories: volume monitoring in combination with forecasts from traffic models [18,19], statistical methods applied to short-period traffic data [20,21], and machine-learning techniques [22,23,24,25,26,27]. In addition, recently, there has been some success in using unconventional data like cellular phone tracking or social media posts to detect incidents [28,29,30], video-based detectors [31,32], and methods based on acoustic data [33].

Distinguishing recurrent and non-recurrent congestion is one of the key areas of research. Nonlinear time series analysis methods developed for complex systems have shown a great potential for distinguishing different types of dynamical behaviour and pinpoint transitions [34,35,36,37,38], but so far their application in the traffic context has been limited [39].

The few examples that developed incident detection by using nonlinear time series analysis methods focused for example on phase space reconstruction [39]. Such techniques are a more compelling way to analyse univariate field data, namely, the method of recurrence plots (RP) [40,41,42] and recurrence quantification analysis (RQA) [37,43]. Recently, based on recurrence plots, an alternative approach has been developed: the Quadrant Scan. Although, the RP method has been widely used for the analysis of time series from fields ranging from finance to physics to biology [34,44,45,46,47,48,49,50,51], Quadrant Scan is a relatively new method developed to detect transitions in system dynamics. It has been successfully applied to time series as well as non-temporal signals. Initially, the Quadrant Scan was introduced in [52] as a tool to identify change points in time series. Later in [53], the idea was further developed to detect ageing in granular materials. In [54,55,56], the method was extended to a weighted scheme and was tested in various applications including synthetic, geological, spectral, and EEG and ECG data. The principles and steps of the Quadrant Scan method have been addressed comprehensively in [54,55].

In this paper, we apply the Quadrant Scan method to traffic volume data related to two incidents. The data is multivariate and results from a multi-sensor system. There are several advantages to using the Quadrant Scan, and we believe it to be a superior tool for such analysis. Unlike the previous analysis that has been conducted on the same data with a univariate RP and RQA implementation of each sensor separately [39], the Quadrant Scan allows a multivariate analysis by integrating the information from multiple sensors. This leads to a more accurate detection in the time–space not only of the incident but also of its consequences (incident end time and queue end time). In addition, the Quadrant Scan provides a concrete and consistent differentiation between the abrupt change and the gradual/smooth change. It allows a successful and strong differentiation between the incident’s effects on the traffic system and normal congestion. Although this discrimination by the Quadrant Scan is quantifiable for an automated decision, in this investigation the shape-based visual analysis is adequate for elaborating the promising capability of the proposed method. Furthermore, the Quadrant Scan is a multi-scale transition detection tool that enables us to detect changes with different impact levels on the traffic flow indicating whether the incident is major or minor. We use one incident dataset to tune and optimise the parameters of the Quadrant Scan. This enables us to further distinguish between different change characteristics of the possible causes (incident or high volume). Accordingly, we use the optimal parameters on the second incident data to verify and validate the performance of the Quadrant Scan in detecting incidents and differentiating between different types of congestion. This allows us to test the transferability of the algorithm from one incident to the other, and therefore we reduce the sample size requirements enormously without any impact on the efficiency of the estimations. The objective of this study is to demonstrate the potential of the multivariate implementation of the Quadrant Scan to accurately detect not only traffic incidents but also their consequences from multi-sensor systems and consistently distinguish them from recurrent congestion, making it an attractive tool for real-world applications.

The remainder of the paper is structured as follows: first we give a description of the data in Section 2. Section 3 outlines the Weighted Quadrant Scan method and provides a guide to effective parameter selection. The results from both incidents using multivariate as well as univariate inputs are reported in Section 4. Section 5 provides a performance comparison with a well–established change–point detection method as well as a method that was used to analyse the same dataset. In Section 6, we discuss our results and highlight the potential for traffic management implementations. Finally, the analysis and outcomes are summarised, and a conclusion is given in Section 7.

## 2. Data

The data used in this study are time series measured by volume sensors on Attika Tollway—a highway in Greece. The Attika Tollway (Attiki Odos) is a two-directional urban motorway, consisting of three lanes and a hard shoulder in each direction. It runs for about 70 km through the northern side of the metropolitan city of Athens from the Elefsina to Sounio. The Traffic Management Centre (TMC) is equipped with cameras and other systems to help the operator, Attikes Diadromes S.A., to monitor traffic, detect incidents and ensure motorists’ safety. Among other sections, the Attika Tollway’s 10 kilometer-long central urban section, usually operates under congested conditions with an average daily traffic of 67,500 vehicles. There were a reported 23,867 handled incidents in 2018, which corresponds to an average of about 65 incidents per day [57].

**Figure 1:**
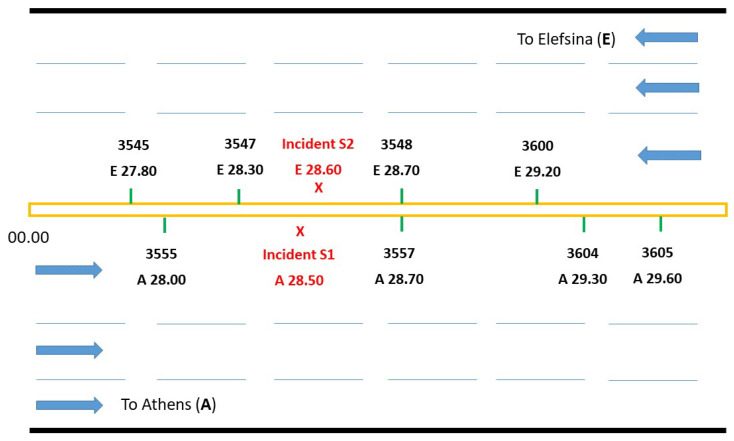
A miniature diagram of Attika Tollway showing the locations of the incidents relative to the positions of the sensors.

A one day (time from 00:00 to 24:00) dataset from eight sensors (four on each side of the freeway) is used as a case study. The sensors record the volume of passing vehicles every 20 s, that is, each sensor profile includes 4320 measurements. Volume data can be used to detect traffic incidents which cause queues that affect normal traffic flow. The data at hand includes two incidents, one in each direction, coded as S1 and S2, respectively, located on the high-speed lanes as shown in Figure 1. The positioning and timing of the two incidents and their consequences are outlined in Table 1. Both incidents occurred on the mainline (not on- or off-ramps), and none of the incidents involved injuries/fatalities. S1 was on the left lane and involved one stalled vehicle owing to mechanical failure. The S1 duration was 44 min with an impact maximum queue length of 2600 m for a 34-min duration. S2 was a rear-end collision between two vehicles, blocking the left lane. The S2 duration was 39 min with an impact maximum queue length of 7000 m for a 114-min duration. A previous analysis has been conducted on this data [39]. The data has been normalised to zero mean and standard deviation of one to facilitate the analysis. A recurrence plot and its quantification analysis were employed to analyse the data of each sensor to detect the incidents. The main characteristic of the univariate analysis was the successful combination of phase space reconstruction methods and algebra on incident detection. For incident S1, the detection was noted by the downstream sensor 3557 and for incident S2 by sensor 3548 (Figure 1). Moreover, reduced speed and increased vehicle density, caused by the accumulated vehicle queue, were located by sensors further from the position of the incident, because incidents S1 and S2 affected the normal traffic flow [39,58]. Table 2 and Table 3 present the identification of the two incidents by the nonlinear methods used during the previous study [39]. In this study, the same set of data will be used, not only for univariate (single sensor) analysis, but also for the multivariate (multiple sensors) analysis employing the proposed Quadrant Scan method. As we will see, the multivariate approach allows data integration and hence more accurate identification of the incident time and its sequences (e.g., queuing, reduced speed, and return to normal flow) and more robust discrimination between recurrent and non–recurrent congestion.

## 3. Methods

**Figure 2:**
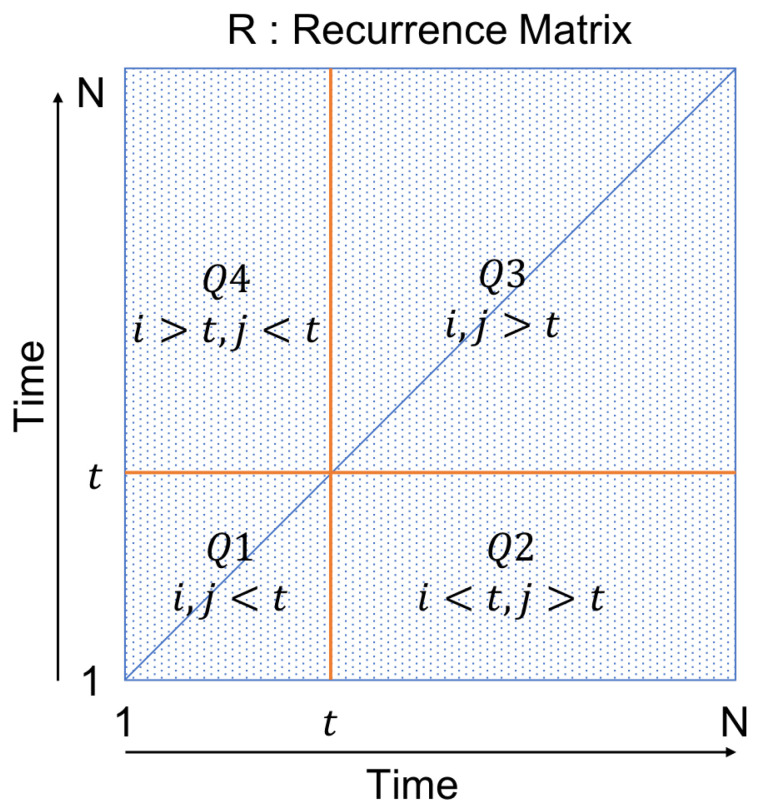
Quadrant Scan principle: at each time index *t*, the recurrence plot is divided into four quadrants. The value of the Quadrant Scan at *t* is evaluated by comparing the recurrence point’s density of quadrants Q1 and Q3 against the point’s density in all quadrants.

In this study, the accident-induced congestion will be visually separated from recurrent congestion. However, further discussion on how this differentiation can be quantified is addressed later in Section 6. Recurrence plots are by now the standard method by which to analyse recurrent features in the field of nonlinear time series analysis, whereas recurrence is the situation in which two states in the phase space of the underlying system get close to each other in different times, and it is a fundamental characteristic of many dynamical systems. A recurrence plot is a powerful visualisation tool. Recurrence quantification analysis (RQA) was developed to analyse and quantify recurrent features in datasets [37]. Our proposed method, the Quadrant Scan method, builds on these methods to detect transitions in a system by scanning its recurrence plots. The Quadrant Scan is an algorithm with the capability to detect change-points in a system’s dynamical behaviour. In simple terms, as demonstrated in Figure 2, the idea of the Quadrant Scan is to divide the recurrence plot at each time index into four quadrants and evaluate the ratio of the point’s density in the first and third quadrants (Q1 and Q3) against the density in all quadrants. There are two versions of the Quadrant Scan, standard and weighted [54,55]. In this traffic application, we use the weighted scheme, which is briefly described below.

### 3.1. Weighted Quadrant Scan

In the scope of the current application, the weighted scheme is comprised of the following three steps.

**Figure 3:**
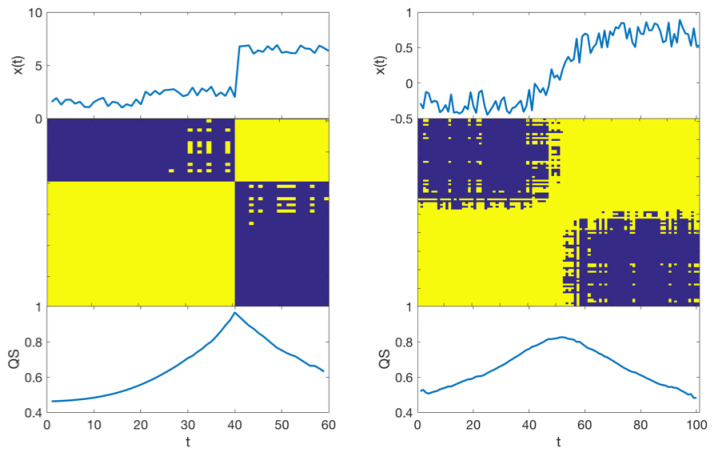
A simulation to demonstrate how the Quadrant Scan visually distinguishes between abrupt and gradual changes. The top panels are the simulated noisy time series, the middle panels are the corresponding recurrence plots (yellow indicates ones and dark blue indicates zeros), and the bottom panels are the resulting Quadrant Scan profiles. The abrupt change is detected as a sharp peak, whereas the gradual change is reflected as a curved peak in the corresponding Quadrant Scan profiles.

1 **Construct a Norm Matrix:** Let $v_{t}^{k}\in R$ be the traffic volume recorded by sensor *k* at time *t*, where k=1,2,⋯,m (if we have *m* sensors), and t=1,2,⋯,N (i.e., there are *N* records). Let $X_{t}=\left(v_{t}^{1},v_{t}^{2},\cdots ,v_{t}^{m}\right)\in R^{m}$ be the multivariate input state at time *t*. We construct a N×N matrix *A*, the entities of which are pairwise distances of states in different times in the phase space of dimension *m*, that is, $a_{ij}=∥X_{i}-X_{j}∥$ for i,j=1,2,⋯,N. Here, ∥·∥ is some norm (distance) measure. In the application of this framework, we use the Euclidean norm.
2 **Construct a Recurrence Plot Matrix:** In this context, we define a recurrence event in the observational data if two states $X_{i}$ and $X_{j}$ are relatively close to each other in the phase space $R^{m}$, that is, the records of the multi-sensor system are relatively similar at these two different time points *i* and *j*. To re-represent the data highlighting the system’s recurrent features in chronological order, a recurrence plot threshold ε is to be adopted, that is, an upper bound distance for which all smaller pairwise distances between the states are counted as recurrences. There are several paradigms to choose the recurrence plot threshold [37,59] (for example, the closest *n* neighbours paradigm which results in a variable ε). To avoid the effect of outliers and minimise the influence of noise in real-world applications, an alternative procedure to select this threshold is proposed in [55] by considering the distribution of the elements of the norm matrix *A* as follows:
  (1) $$\varepsilon =\alpha \times (\mu \left(a_{ij}\right)+3\times \sigma \left(a_{ij}\right)),\mathrm{for}\;\mathrm{all}\;i\;\mathrm{and}\;j\;\mathrm{where}\;0<\alpha <1$$
  where $\mu (a_{ij})$ and $\sigma (a_{ij})$ refer respectively to the mean and standard deviation of the distribution of the elements of *A*. To reduce the effect of outliers, this equation only considers the distance matrix entries within 3 standard deviations away from the mean to select the recurrence plot threshold.
  Then, from *A*, we construct a N×N binary matrix *R* with elements
  (2) $$r_{ij}=\left\{\begin{array}{cc} 1; & a_{ij}<\varepsilon \\ 0; & \mathrm{otherwise} \end{array}\right.$$
  where the ones reflect the recurrence points corresponding to different times in the time space and the zeros reflect no recurrence at the corresponding time points.
3 **Extract Weighted Quadrant Scan (*WQS*) profile:** At each time index *t*, we estimate the Weighted Quadrant Scan value WQS(t) from the recurrence plot matrix *R*, and some weighting matrices as follows (see [55]):
  (3) $$WQS\left(t\right)=\frac{\sum Q1\circ \frac{W1}{w1_{t-1,t-1}}+\sum Q3\circ \left(\frac{W3}{w3_{1,1}}\right)}{\sum Q1\circ \frac{W1}{w1_{t-1,t-1}}+\sum Q3\circ \frac{W3}{w3_{1,1}}+\sum Q2\circ \frac{W2}{w2_{t-1,1}}+\sum Q4\circ \frac{W4}{w4_{1,t-1}}}$$
  where ∘ is the element–wise multiplication and Σ is the sum of the entire elements of the matrix. Qk for *k* = 1, 2, 3 and 4 are the four quadrant sub-matrices from the recurrence plot matrix which are defined at each time index *t* as follows:
  $$\begin{array}{ccc} Q1 & = & (r_{ij})\;\mathrm{for}\;i,j<t\\ Q2 & = & (r_{ij})\;\mathrm{for}\;i<t,j>t\\ Q3 & = & (r_{ij})\;\mathrm{for}\;i,j>t\\ Q4 & = & (r_{ij})\;\mathrm{for}\;i>t,j<t \end{array}$$
  Wk for k= 1, 2, 3 and 4 are four weighting matrices defined at each time index *t* by
  $$\begin{array}{ccc} W1 & = & V1^{T}\times V1\\ W2 & = & V1^{T}\times V2\\ W3 & = & V2^{T}\times V2\\ W4 & = & V2^{T}\times V1. \end{array}$$
  where V1 and V2 are two weighting vectors defined by using the hyperbolic tangent function as follows:
  (4) $$\begin{array}{ccc} V1(l_{1}) & = & \frac{1}{2}\left(1-\tanh\left(\left(\left(t-l_{1}\right)-m_{1}\right)/m_{2}\right)\right) \end{array}$$
  (5) $$\begin{array}{ccc} V2(l_{2}) & = & \frac{1}{2}\left(1-\tanh\left(\left(l_{2}-m_{1}\right)/m_{2}\right)\right), \end{array}$$
  where $l_{1}=1\cdots t-1$, $l_{2}=1\cdots N-t$ and $m_{1}$, $m_{2}\in R$ are two parameters used to tune the smoothness of the weighting scheme. The role and sensitivity of these parameters are discussed in the following subsection.
  Finally, $wk_{i,j}$ refers to the i,j element of the *k* weighting matrix. To normalise the weights between 0 and 1, the weighting matrices are divided by the largest entry of each one.
  Briefly, the weighted version of Quadrant Scan gives larger weights, i.e., larger impact, to the points that are closer to the indexed point in the time space while these weights decline gradually to reach zero as the points are getting farther in the time space (i.e., the farther points in the time space have less impact on evaluating the Quadrant Scan value).
  There are several reasons behind proposing the Quadrant Scan to tackle the incident detection problem. It is able to use multivariable input, that is, multi-sensor input. It is also able to distinguish between abrupt and gradual changes. That is, it has the potential to distinguish between traffic incidents which cause sudden changes in the traffic flow and the recurrent traffic congestion which builds up gradually. In a previous work [55], we show that the Quadrant Scan curve demonstrates sharp peaks which reflect abrupt changes in the dynamics of the system under consideration. However, if the system dynamics change gradually, the Quadrant Scan identifies such changes as curved peaks (Figure 3, bottom panels). This is a result of the fact that abrupt changes of a system are reflected by bands of zeros in the structure of the corresponding recurrence plot whereas drifts (gradual transitions) are reflected by fading to the upper left and lower right corners [37] (Figure 3, middle panels). Consequently, the Quadrant Scan is a suitable method that analyses the traffic volume’s recurrence plot to detect traffic incidents and differentiate them from recurrent congestion.

### 3.2. Parameter Settings

The Weighted Quadrant Scan algorithm requires the setting of three parameters given that no embedding has been done for the multivariate application in this framework. In [55] a guide for potential users on how to set up the method’s parameters was provided. In general, these parameter settings are problem-dependent, and so here we elaborate upon some facts that help to better understand the roles of these parameters on the method’s performance to allow better selection strategy. The first parameter to discuss is the recurrence plot threshold ε. In fact, there are two main procedures to set up this threshold: a constant mass (the same number of neighbours for each time point) or a constant volume or density of points for the entire recurrence plot [37]. Although the original Quadrant Scan scheme was proposed to use a constant mass threshold (fixed number of neighbours), to allow multiple-scale detection and reduce the noise effect in our application, we consider a constant volume principle by using the scale factor α and the norm matrix distribution as defined in Equation (1). As demonstrated in [55], an ordinal variation from larger to smaller values of α allows the detection of behavioural transitions at different scales. In the traffic application, because incidents may have different levels of impact on the traffic flow—e.g., major incident or minor incident—different settings of this parameter allow for the detection of different impact levels, that is, a larger value of α allows the detection of incidents with only higher impact, whereas a smaller value of α allows the detection of incidents with higher and lower impact. This merit will be discussed further in Section 6. The second and third parameters are the weighting parameters $m_{1}$ and $m_{2}$ which determine the sharpness or smoothness of the weighting schemes. As demonstrated in [55], the Quadrant Scan performance is not very sensitive to relatively small deviations of these weighting parameters albeit tuning them is problem-dependent. In general, there are three schemes of weightings, $\left(m_{1},m_{2}\right)=\left(200,50\right),\left(100,25\right)$ or (50,10). The first scheme considers a wider window around the indexed point of past and future points with higher weight (i.e., impact) in the calculation of the Quadrant Scan value. For this scheme, approximately the first 100 time points around the indexed time point in both past and future have a full contribution in evaluating the Quadrant Scan value. Then the contribution of the farther time points decreases gradually to reach a zero contribution for the time points that are ≈300 time steps away from the indexed point. The second and third schemes have, respectively, a narrower impact window with a sharper decreasing rate around the indexed point as the base to decide whether there is a change in the dynamics (for more details see [55]). In terms of traffic, the minimum data length and time tolerance that are required for real-time detection employment can be determined by testing the different weighting schemes.

## 4. Results

The approach of analysing these data and validating the consistency of the proposed method is as follows. There are two multi-sensor datasets of the same system representing two different incidents. The method includes three parameters which require tuning (depending on the problem) to allow the method to successfully satisfy the objective of the analysis, that is, optimal incident detection and successful separation from recurrent congestion by integrating multiple sensors’ information. In the proposed analysis paradigm, one multi-sensor incident data (S1) is used to explore different settings of the parameters to investigate the detection method performance and the range of parameters that allows a differentiation between the abrupt changes caused by incidents and the gradual congestion caused normally in rush hours. Consequently, to verify the performance of the method, the selected parameter setting is used on single sensors of the same incident. Furthermore, to validate the consistent performance of the method on the considered traffic system, we test the method performance on the second incident dataset (multivariate and univariate analysis) by using these optimized parameters.

### 4.1. Incident S1

Incident S1 data is used to optimize the algorithm parameters, investigate the method capability to detect changes in the system dynamics, and in particular, identify those caused by incidents and distinguish them from the changes caused by normal traffic congestion. A multivariate signal using all sensors’ profiles is analysed under several parameter settings to select an appropriate setting that allows incident detection and differentiation from normal congestion. Afterwards, univariate analyses was done to verify the performance quality.

#### 4.1.1. Multivariate Analysis—Multiple Sensors

**Figure 4:**
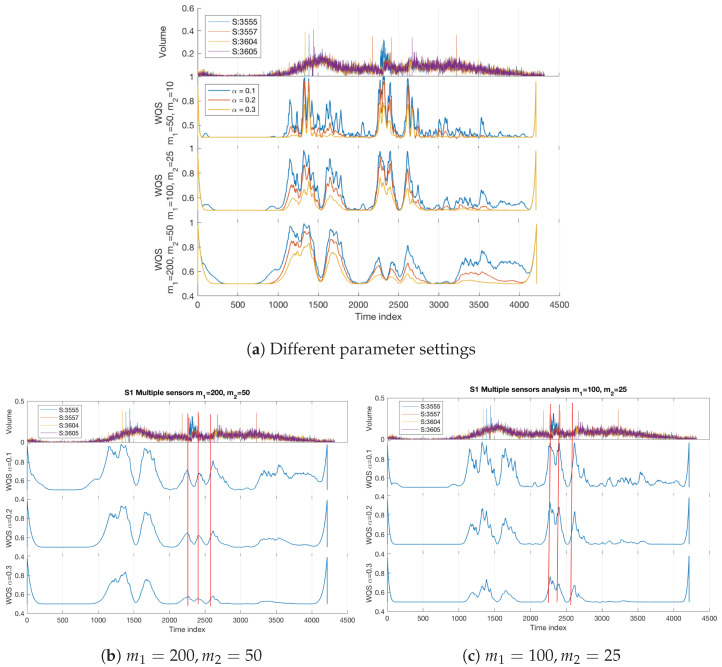
Incident S1 multiple sensor analysis. In panel (**a**), different parameter settings are investigated. In panels (**b**,**c**), the vertical red lines refer to the incident start time, incident end time, and queue end time, respectively (see Table 1).

In this analysis, we utilise the S1 multivariate data from the four different sensors (i.e., m=4) to investigate different settings of the parameters in order to choose a weighting scheme and appropriate scale for an objective detection. As discussed above in Section 3.2, three weighting schemes ($\left(m_{1},m_{2}\right)=\left(200,50\right),\left(m_{1},m_{2}\right)=\left(100,25\right),\left(m_{1},m_{2}\right)=\left(50,10\right)$) over three different scales (α=0.1,0.2,0.3) are considered. The results shown in Figure 4 demonstrate the ability of the Quadrant Scan to detect the transitions in the traffic system by using a multivariate input from multiple sensors. Panel (a) shows that the narrow and sharp weighting scheme for $\left(m_{1},m_{2}\right)=\left(50,10\right)$ fails to distinguish between dynamical changes caused by the incident and those caused by rush hour congestion whereas the other two wider and smoother weighting schemes succeed ($\left(m_{1},m_{2}\right)=\left(200,50\right),\left(m_{1},m_{2}\right)=\left(100,25\right)$). Panels (b) and (c) show that the Quadrant Scan successfully detected the incident start time, the incident end time and the queue end time, which are given by Table 1 and indicated as vertical red lines. It is observable how the Quadrant Scan’s sharp peaks match these red lines. Moreover, these changes related to the incident are reflected in the Quadrant Scan profiles as sharp peaks whereas the gradual changes caused by the normal congestion at rush hours (between time indices 1000 and 2000) are reflected as curved peaks. The results indicate a relatively better performance for the weighting parameters ($\left(m_{1},m_{2}\right)=\left(100,25\right)$). This is particularly true in detecting the times of the incident and its consequences (see in Figure 4 panels (b) and (c) how the sharpness of the peaks matches the red vertical lines) although the curvature level for the peaks at the normal gradual congestion is more observable in panel (b). However, this is due to recording failure of some sensors at this period, a point that will be elaborated upon further in the next subsection. Selecting $\left(m_{1},m_{2}\right)=\left(100,25\right)$ for the weighting scheme means that the non-recurrent incidents for the considered traffic stream are detectable and distinguishable for a window of the sensors’ observations with a radius in the range of 100 to 150 time points from the incident point. Consequently, this weighting scheme will be considered for the rest of the analysis for validation and verification.

#### 4.1.2. Univariate Analysis—Single Sensor

**Figure 5:**
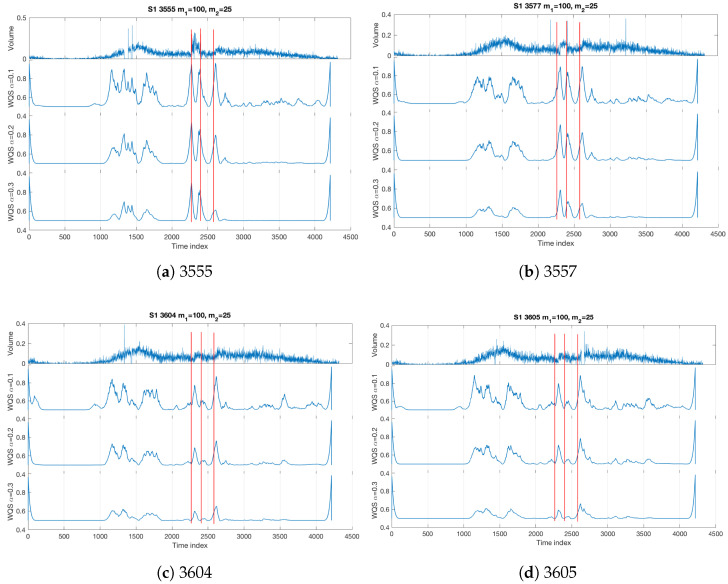
Incident S1 single sensor analysis. The weighting parameters are $m_{1}=100,m_{2}=25$ over different recurrence plot threshold scales α=0.1,0.2,0.3. The vertical red lines refer to the incident start time, incident end time, and queue end time, respectively (see Table 1).

After the successful detection using multivariate data from all the sensors, we investigate the performance of the Quadrant Scan when single sensor data is used individually. To validate the consistency of the performance, the same weighting scheme ($\left(m_{1},m_{2}\right)=\left(100,25\right)$) is used. Figure 5 show the results obtained from each sensor. In panel (a), one can observe how Sensor 3555 allowed an accurate detection of the changes related to the accident (see how the sharp peaks match the incident time and its consequences which are indicated by the vertical red lines) as well as a visual differentiation from the curved peaks caused by the recurrent congestion (time indices between 1000 and 2000). Sensor 3557 (panel b) allowed the detection of the changes related to the incident but with some time delay because of the location of the sensor relative to the incident location. It is a worthwhile observation that the sharp peaks and curved peaks are more distinguishable here than in sensor 3555 (and other sensors). In fact, the discrimination is more observable as the data of this sensor during the period of the recurrent gradual congestion does not show any dropping or missing data due to the sensor’s recording failure, unlike for sensor 3555, which shows obvious recording failure within this period (see Figure 5a just before time index 1500). Sensors 3604 and 3605 (panels c and d) were less efficient in detecting all the changes caused by the incident. The incident start time was detected with a longer delay, the incident end time was not detected properly while the queue end time was detected. The reason why the incident end time was not detectable by these two sensors is that the dynamic transition scale level is relatively close to the noise level. It is necessary to emphasise here that all sensors allowed the detection of the rush hour congestion as curved peaks unlike the sharp peaks which reflect the abrupt changes related to the incident.

### 4.2. Incident S2

Incident S2 data is used to validate the consistency of the method’s performance by testing the parameter settings selected by incident S1 data on a different dataset from the same system. Again, the Quadrant Scan performance is investigated by using multiple sensor data as well as single sensor data.

#### 4.2.1. Multivariate Analysis—Multiple Sensors

**Figure 6:**
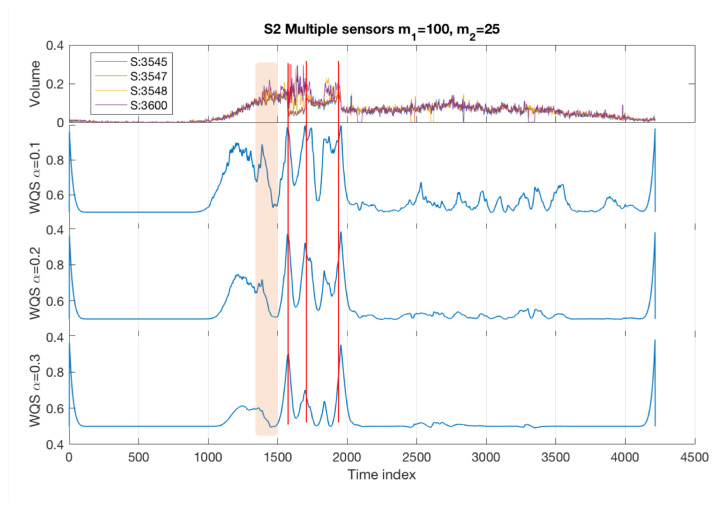
Incident S2 multiple sensor results with weighting parameters $m_{1}=100,m_{2}=25$ over different recurrence plot threshold scales α=0.1,0.2,0.3. The vertical red lines refer to the incident start time, incident end time and queue end time respectively (see Table 1). The shaded area highlights an incident with a lower impact level on the traffic flow than the major incident S2—this was only detected by the smaller value of α.

In this analysis, we take into account the information collected from all four sensors and use a multivariate input. We consider the selected weighting parameters ($m_{1}=100$, $m_{2}=25$) over different scales (α=0.1,0.2,0.3). The results are shown in Figure 6 in which the Quadrant Scan successfully detected all the changes in the system’s dynamical behaviour caused by the accident. This is shown as the sharp peaks match the vertical red lines which indicate, respectively, the incident start time, the incident end time, and the queue end time given in Table 1. Moreover, the Quadrant Scan visually distinguishes between these abrupt changes (as sharp peaks) and the gradual changes caused by the recurrent congestion (curved peak between time indices 1000 and 1500). This validates the method’s performance and its capability to detect the changes in the traffic system’s dynamical behaviour and to differentiate the changes caused by incidents from changes building up gradually under dense traffic.

#### 4.2.2. Univariate Analysis—Single Sensor

**Figure 7:**
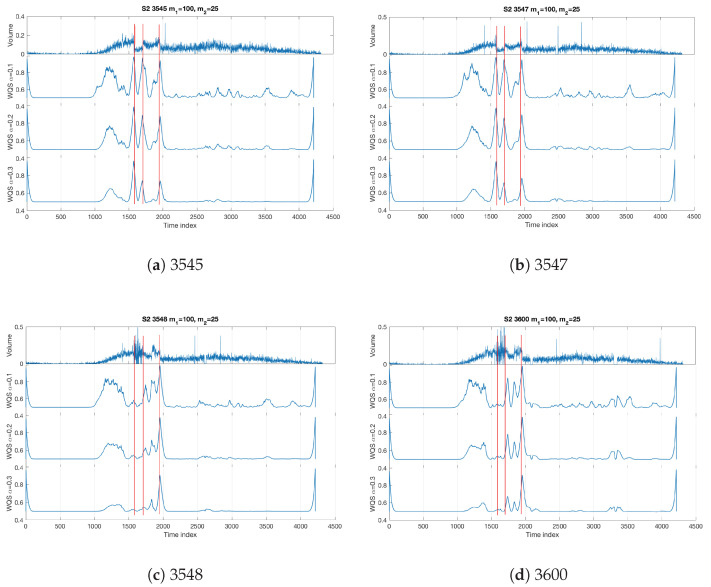
Incident S2 single sensor analysis. The weighting parameters are $m_{1}=100,m_{2}=25$ over different recurrence plot threshold scales α=0.1,0.2,0.3. The vertical red lines refer to the incident start time, incident end time, and queue end time respectively (see Table 1).

Now we consider the single sensor datasets separately and investigate the Quadrant Scan’s performance on the selected weighting parameters ($m_{1}=100,m_{2}=25$) over different recurrence plot threshold scales (α=0.1,0.2,0.3). The results shown in Figure 7 infer that sensors 3545 and 3547 successfully detect all of the changes in the traffic system caused by the incident; the sharp peaks match the vertical red lines which indicate the times of the incident and its consequences. However, the sensors 3548 and 3600 fail to detect the incident start time by using the selected parameters (this will be discussed in the following Section 6), whereas the incident end time was detected with some delay and the queue end time was better detected. One can observe how the results from all sensors discriminate the sharp peaks and the curved peaks which confirm the Quadrant Scan ability to distinguish the changes in traffic flow caused by an incident and the changes owing to recurrent congestion.

## 5. Comparison with Other Methods

In this section, we compare the proposed multivariable Quadrant Scan method with: (1) the well-established Wavelet Transform (WT) method that is well-known as a robust tool for change–point detection [60]; and (2) the previous nonlinear time series and phase space reconstruction-based method that was proposed to analyse the same dataset in [39].

### 5.1. Comparison with WT

**Figure 8:**
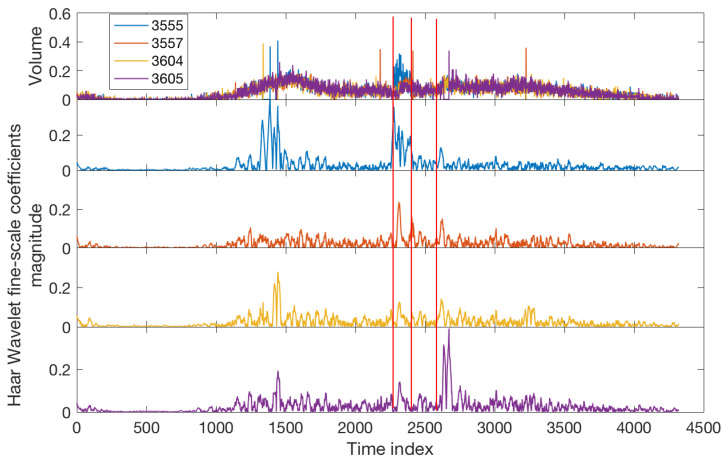
Incident S1: Analysis of Haar Wavelet Transform. Top panel is the volume data from four different sensors. The lower panels are the analysis results from sensors 3555, 3557, 3604, and 3605 respectively. The vertical red lines refer to the incident start time, incident end time, and queue end time respectively (see Table 1).

**Figure 9:**
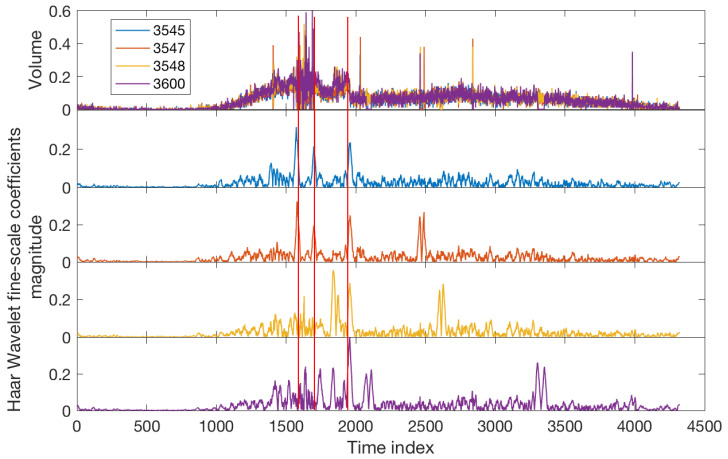
Incident S2: Analysis of Haar Wavelet Transform. Top panel is the volume data from four different sensors. The lower panels are the analysis results from sensors 3545, 3547, 3648, and 3600, respectively. The vertical red lines refer to the incident start time, incident end time, and queue end time, respectively (see Table 1).

For this benchmark, we use the Haar WT and plot the coefficient’s magnitude for a fine scale. The Haar WT is a well-known tool for the analysis of signals with abrupt transitions [61]. It is applicable for univariate time series; therefore, the detection analysis for each sensor is conducted individually. The results for incidents S1 and S2 are shown in Figure 8 and Figure 9 respectively. Comparsion with the corresponding results of the proposed WQS (Figure 4 and Figure 6) shows the superiority of the new method on several levels. Firstly, the integration of information that is allowed by the WQS leads to more accurate results; this is confirmed by Table 4 which compares the performance of the methods for detecting the incident’s start time. Although the performance of WT for incident S2 and its consequences is only slightly less accurate than the WQS (Figure 9 and Table 4), it fails to detect S1 and its consequences as accurate as the WQS, specifically the incident’s start time and the queue’s end time (see Table 4 and Figure 4 panels b and c against Figure 8). Secondly, in terms of traffic management, the merit of data integration provides a more efficient monitoring strategy as it only requires monitoring one integrated profile for each multi-sensor system rather than monitoring each sensor separately. A third notable advantage of WQS is that it allows discrimination of different congestion types (see in Figure 4 and Figure 6 the difference between the curved peaks which are related to recurrent peak hours congestion and sharp peaks which are related to the incidents) whereas the WT analysis fails to distinguish different congestion types (see the analysis results in Figure 8 and Figure 9 at the periods of the recurrent peak hours congestion). For S1, WT shows similar peaks to those resulting from the incident period whereas for S2, WT fails to detect the gradual recurrent build–in congestion. Finally, the WT shows more false alarms than the WQS. For example, see the false peaks from the different sensors after time index point 2500 in Figure 9 whereas such false positives are not present in the WQS analysis (Figure 6).

### 5.2. Comparison with a Previously Used Phase Space Reconstruction-Based Method

The comparison with the previously used nonlinear time series analysis and phase space reconstruction-based method to analyse the dataset demonstrated the benefits of the proposed Quadrant Scan scheme. This is evidenced in two perspectives: first, accuracy-wise, the Quadrant Scan is clearly superior in detecting the accurate times of both incidents and their consequences (see Figure 4 and Figure 6). The previous methodology detected less accurately the times of the incidents (Table 4). Secondly, efficiency-wise, the Quadrant Scan allows discrimination of incident-induced congestion from recurrent congestion as well as data integration from multiple sensors whereas the previous method was restricted to univariate data to detect the incident’s time, which makes in practice the Quadrant Scan more efficient for real-world applications.

## 6. Discussion

In this section, we discuss and explain a number of points observed by the analysis results. (1) The first observation is that the single sensor analysis failed on some occasions (based on the incident time and location relative to the sensor’s location) whereas the combined multivariate analysis allowed an accurate and consistent detection and differentiation of the congestion types. This reflects the advantage of using a tool that allows the integration of information from multivariate signals. (2) The second observation is that in incident S1, the sensor upstream the incident site (sensor 3555) performs better (Figure 5). However, in the case of S2, the sensors downstream from the incident site (sensors 3545 and 3547) perform better (Figure 7). This is because incident S2 occurred while the traffic volume was already dense; therefore the sensors downstream of the incident indicate a sharp drop in the volume data. On the contrary, S1 did not occur under already congested conditions, that is, it was recorded when the traffic’s volume was low, therefore sensors upstream the incident indicate a sharp jump in the volume data. (3) In incident S1, the incident end time was not detectable by sensors 3604 and 3605 because of the time and relative location of the incident which caused a low level of change in the traffic dynamics. This change was relatively small and close to the noise level associated with the data which restrains the ability of the detection. However, as shown in Figure 5, using different scales of the recurrence plot threshold indicates that smaller thresholds can detect such change but the smaller the threshold the more likely to produce other peaks caused by the random noise in the data and not relevant to any incident. (4) The merit of using α as a scalar parameter for multi-scale detection to detect incidents with different impact levels (i.e., major and minor incidents) is observable in the analysis of incident S2 (Figure 6). At the time index just before 1500 (the shaded area), the signal shows a change of a lower level than the change caused by the major incident S2. This could be due to a minor incident with less impact. This lower-impact incident was detected for a smaller α=0.1, but not detected for a larger α=0.3. The major incident S2 was detected for all the values of α reflecting the higher impact of such incident. (5) In incident S2, the incident start time was not detectable by sensors 3548 and 3600 although the relevant volume signals show a change in the amplitude. This is because the Quadrant Scan scheme used in the analysis aimed to detect state transitions rather than dynamic transitions, and to detect this type of dynamical transition requires an appropriate embedding to the signal [55,59,62]. However, using a multivariate signal by combining the information from all sensors allows an accurate detection. (6) The comparison with the well-known change–point detection Wavelet Transform method and the phase space reconstruction-based method that is previously used to analyse the dataset demonstrated the advantages and merits of using the proposed Quadrant Scan scheme. The accuracy and efficiency level of the WQS that is achievable by integrating the information from multiple sensors as well as the capability of distinguishing recurrent congestion from non-recurrent congestion due to incidents make the WQS in practice a more efficient tool for real-world applications. (7) Finally, it is noteworthy here that if a sudden or abrupt change occurs in the traffic system but not because of an accident, the Quadrant Scan method would still detect that as a sharp peak. This limitation is due to the fact that the Quadrant Scan does not require any prior or further information other than the traffic volume signals. Therefore, any accident-like impact on the traffic flow is detected as a sharp peak.

## 7. Conclusions

In this study, the recurrence-based Quadrant Scan methodology has been proposed as a tool to analyse traffic volume data to detect incidents with an impact on the traffic flow by integrating multiple sensors’ data. As we have seen in the discussion, multiple sensor data is not only useful, but also important for the detection of incidents in different traffic conditions. With respect to traffic incident management, the proposed algorithm shows several important implications. First, it is applicable to a multi-sensor system which avoids the added workload of monitoring the signal of each sensor separately. Moreover, integrating the information from all of the sensors enables more accurate detection and discrimination of the incidents. Second, the method is capable of distinguishing between the non-recurrent congestion caused by an incident from recurrent congestion during peak hours. This results from the fact that the Quadrant Scan differentiates between gradual changes (recurrent congestion) and abrupt changes (non-recurrent congestion) by projecting the former as curved peaks and the latter as sharp peaks. Although this discrimination has been investigated visually through this study as proof of the concept, it can be quantified by measuring the width and the gradient of the peaks. This will be the subject of future work when more data is involved. In this manuscript, we investigated and validated the potential of the Quadrant Scan as a promising tool for this task. Furthermore, the method successfully detects incidents during heavy traffic and recurrent congestion (as shown in incident S2). Third, the method is able to detect the consequences of an incident, that is, it detects the incident’s end-time and the queue’s end-time before normal flow restoration. Fourth, the proposed method allows multi-scale detection by varying the parameter α which enables the discrimination between major incidents and minor incidents those have different impact levels on the traffic flow. Finally, the analyses verify that the method is transferable and performs consistently among different incidents which minimises the cost of training and tuning. In fact, one merit of the QS method is that it is an unsupervised tool that does not require prior information about the traffic system. Therefore, any type of data that describes the traffic dynamic or characteristics would be applicable to the QS tool to detect incidents. Although some other sources (e.g., traffic speed or integration of different characteristic sources) could be better indicators for traffic disruptions than the traffic volume, the success of QS to accurately detect the incidents from only the traffic flow volume signal shows the robustness of the method. However, this unsupervised nature of the QS could arise in practice as a limitation. That is, any incident-like impact (i.e., abrupt change or disruption) on the traffic flow which can also result from other causes than accidents, could be lead to a sharp peak in the QS. Given this potential for false positives, further experiments on a larger-scale dataset are needed to investigate the false positive rate as well as to fine tune the scale and weighting parameters of the WQS to overcome this limitation or at least make the false positive as rare as possible. This is a direction for a future extension.

Our outcomes demonstrate that the Quadrant Scan method can be applied to real-time incident detection with a short delay. Due to the limitation of our current dataset, other types of non-recurrent congestion were not tested, which will be a promising direction for future research.

## Figures and Tables

**Table 1:** Positioning and timing of the incidents and their consequences as reported from the Traffic Management Centre (TMC).

Incident	Position	Date	Incident Start Time	Incident End Time	Queue End Time
S1	A 28.5	01/03/2010	12:35 (2268)	13:19 (2402)	14:19 (2581)
S2	E 28.6	02/03/2010	8:48 (1587)	9:27 (1706)	10:45 (1940)

**Table 2:** Incident S1 (position: A 28.5, true time point index: 2268, true time 12:35:40): The detection results obtained by each sensor using the previous phase space reconstruction–based method [39].

Incident S1		
Sensor Position Upstream (Up)	Sensor ID	Time Series Point of Incident Detection
A 28.00 (Up)	3555	2240 (time 12:26:20)
A 28.70 (Dn)	3557	2251 (time 12:30:00)
A 29.30(Dn)	3604	2543 (time 14:07:20)
A 29.60 (Dn)	3605	2566 (time 14:15:00)

**Table 3:** Incident S2 (position: E 28.6, true time point index: 1587, true time 8:48:40): The detection results obtained by each sensor using the previous phase space reconstruction–based method [39].

Incident S2		
Sensor Position Upstream (Up)	Sensor ID	Time Series Point of Incident Detection
E 27.80 (Dn)	3545	1872 (time 10:23:40)
E 28.30 (Dn)	3547	1872 (time 10:23:40)
E 28.70 (Up)	3548	1524 (time 8:27:40)
E 29.20 (Up)	3600	1536 (time 8:31:40)

**Table 4:** Comparison of the performance of the three different methods for detecting the incident start time. The absolute time difference between the actual reported time by the Traffic Management Centre (TMC) and the detected time from the multivariable WQS, WT from the best sensor and the phase space reconstruction–based methods are outlined.

The Absolute Time Difference between the Actual Reported Time by the TMC and the Detected Time			
Incident	Multivariable WQS	WT	The Phase Space Reconstruction–Based Method
S1	40 s	160 s	340 s
S2	240 s	280 s	1020 s

## Data Availability

The data that support the findings of this study are available on request from the corressponding author, [A.Z.].

## Abbreviations

The following abbreviations are used in this manuscript:

TT	Travel Time
TMC	Traffic Management Centre
S1	Incident 1
S2	Incident 2
RP	Recurrence Plot
RQA	Recurrence Quantification Analysis
QS	Quadrant Scan
WQS	Weighted Quadrant Scan
WT	Wavelet Transform
EEG	Electroencephalography
ECG	Electrocardiography
